# Evaluating the interactions between vibrational modes and electronic transitions using frontier orbital energy derivatives†

**DOI:** 10.1039/d4cc02066a

**Published:** 2024-07-02

**Authors:** Lisa A. Schröder, Harry L. Anderson, Igor Rončević

**Affiliations:** a Department of Chemistry, Oxford University, Chemistry Research Laboratory Oxford OX1 3TA UK igor.roncevic@chem.ox.ac.uk; b Institute of Physical Chemistry, Karlsruhe Institute of Technology, KIT Campus South Fritz-Haber-Weg 2 D-76131 Karlsruhe Germany

## Abstract

Vibrations affect molecular optoelectronic properties, even at zero kelvin. Accounting for these effects using computational modelling is costly, as it requires many calculations at geometries distorted from equilibrium. Here, we propose a low-cost method for identifying vibrations most strongly coupled to the electronic structure, based on using orbital energy derivatives as a diagnostic.

## Abstract 2

Frontier orbital energy derivatives can be used to identify vibrations strongly coupled to electronic structure.

## Graphical abstract

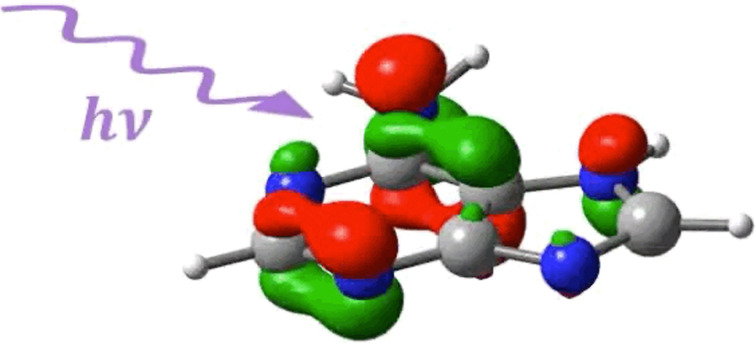

Predicting the barriers to electronic excitation and charge transfer is essential for engineering materials for solar cells,^1^ mimicking redox processes in enzymes,^2^ and building molecular electronic devices.^3^ Advances in electronic structure theory have enabled accurate descriptions of electronic states and the couplings between them,^4–7^ facilitating *in silico* design and screening of potential next-generation molecular devices. However, most work on predicting molecular properties such as vertical excitation energies, polarizabilities, or single-molecule conductances is limited to equilibrium geometries, ignoring effects of nuclear motion.^4,5,8,9^ This approach works well when the coupling between electronic states and nuclear motion is small and symmetric. In other words, if movement in direction +*Q* and −*Q* (which can be represented as a linear combination of normal modes *Q*_*i*_) changes some property by a small amount +*δ* and −*δ*, respectively, the effect of nuclear motion can be approximated by a mere broadening (proportional to d*δ*/d*Q*) of the observed property.

Recently, Bai *et al.*^10^ demonstrated that this approximation does not always hold by showing that computed vertical excitation energies usually shift to lower energies when effects of nuclear motion are included. Alvertis and collaborators^11^ found that this gap renormalization is present even at zero kelvin (zero-point renormalization, or ZPR), and estimated its mean value to −0.35 eV for Thiel's set^4^ of small organic molecules, with extreme cases reaching −1.36 eV. They also noted that ZPR can often be reduced to the effect of only several vibrations. A similar conclusion was reached by Lambropoulos *et al.*, who found that ZPR in cyclo[18]carbon can be reduced to the effect of a few bond-stretching vibrational modes.^12^ In organic molecular wires, bond stretching and backbone torsion play a large role, as the charge-carrier mobilities are highly dependent on the extent of conjugation modulated by these vibrations.^13–15^ In more general cases, it can be challenging to understand which vibrations are important and which can be neglected.

Here, we provide a straightforward, inexpensive method for investigating the effect of individual normal modes on the electronic structure, based on calculating derivatives of frontier orbital energies with respect to molecular vibrations. We first use this method to evaluate how vertical excitation energies are modulated by specific vibrations, and then we analyse the effect of specific vibrations in a system with strong electron-vibration coupling.

In the framework of time-dependent density functional theory (TD-DFT), the first optical transition can usually be attributed to an excitation of a single electron from the highest occupied molecular orbital (HOMO) to the lowest unoccupied molecular orbital (LUMO). The energy difference between the HOMO (*E*_H_) and the LUMO (*E*_L_) can be taken as a measure of the fundamental gap (*E*_gap_), which is more rigorously defined as the difference between the ionization potential and the electron affinity.^16^ At the equilibrium geometry *Q*_eq_ = 0, the first vertical excitation energy (*E*_S1_; also called the optical gap) will differ from the fundamental gap (*E*_gap_) by the exciton binding energy (*E*_bind_):

1 *E*_S1_(*Q*_eq_ = 0) = *E*_gap_(0) − *E*_bind_(0) = *E*_L_(0) − *E*_H_(0) − *E*_bind_(0)

In contrast to *E*_gap_, which is a one-electron quantity (difference between orbital energies), *E*_bind_ includes the two-body Coulombic attraction between the newly formed electron–hole pair. When the geometry is displaced along a normal mode *i* by some amount by *Q*_*i*_, the optical gap changes by:

2 Δ*E*_S1_(*Q*_i_) = [*E*_L_(*Q*_i_) − Δ*E*_L_(0)] − [*E*_H_(*Q*_i_) − Δ*E*_H_(0)] − [*E*_bind_(*Q*_i_) − Δ*E*_bind_(0)]

which can also be written as:

3 Δ*E*_S1_(*Q*_i_) = Δ*E*_gap_(*Q*_i_) − Δ*E*_bind_(*Q*_i_)

In practice, the effect of nuclear motion on the S_1_ energy Δ*E*_S1_ is determined by performing a series of excited-state calculations on geometries displaced by *Q*, which can either be obtained by Monte Carlo sampling (50–100 samples, in which *Q* for each geometry is a random linear combination of *Q*_*i*_) or by finite differences (which usually involves displacements by +*Q*_*i*_ and −*Q*_*i*_ for each normal mode *i*). However, if the exciton binding energy is much less sensitive to geometric changes than frontier orbital energies (*i.e.*, Δ*E*_bind_ ≪ Δ*E*_gap_), then the changes in the optical gap due to nuclear motion Δ*E*_S1_ can be approximated by the change in the fundamental gap Δ*E*_gap_. We call this neglect of the geometry dependence of the two-body terms (Δ*E*_S1_ ≈ Δ*E*_gap_) the static binding approximation (SBA). SBA draws from early work on qualitative MO theory,^17^ and it is commonly used the popular Hubbard–Peierls model (see ESI†).^18^ SBA is attractive for two reasons. One, it simplifies the evaluation of effects of nuclear movement. When the harmonic approximation is valid (small *Q*), Δ*E*_gap_ can be calculated from derivatives of orbital energies with respect to normal modes, d*E*_H_/d*Q*_i_ and d*E*_L_/d*Q*_i_, which can be obtained in a single ground-state calculation.^19^ Two, the SBA enables us to identify the vibrations most strongly coupled to the electronic structure from the magnitude of their orbital energy derivatives with respect to nuclear movement.

To investigate how well the static binding approximation holds, we tested it on the Thiel's set of small organic molecules (Fig. 1), for which the ZPR was investigated in ref. 11. We computed Δ*E*_gap_ using orbital energy derivatives and compared it to Δ*E*_S1_ obtained using TD-DFT (B3LYP^20^/def2-SVP), calculated for geometries modulated along each normal mode *Q*_*i*_ by the standard deviation of the thermal distribution *σ*_*i*_(*T*):^11^4
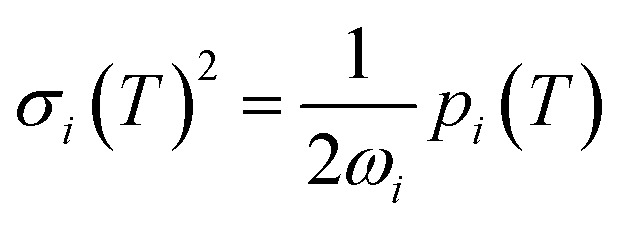
where *ω*_*i*_ is the vibrational frequency, *T* is the temperature, and *p*_*i*_(*T*) is the Bose–Einstein population at *T*, equal to unity at *T* = 0. Eqn (4) gives us a way to account for zero-point vibrations by looking at only two characteristic geometries modulated from the equilibrium by ±*σ*_*i*_(0), as shown by Hele *et al.*^11^

**Fig. 1:**
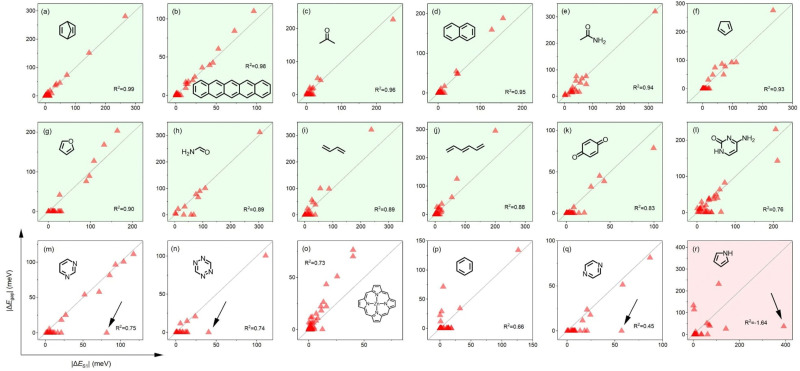
Electronic-vibrational coupling calculated using Δ*E*_gap_ (vertical axis) and Δ*E*_S1_ (horizontal axis; both in meV) for selected molecules from Thiel's set, decomposed by normal modes (triangles) and evaluated for zero-point vibrations. Black arrows in (m), (n), (q) and (r) correspond to stretching vibrations with a large Δ*E*_S1_ relative to Δ*E*_gap_. Results for remaining molecules are given in Fig. S1 (ESI†).

We find that SBA holds reasonably well across Thiel's set (Fig. 1 and Fig. S1–S3, ESI†): in 18 out of 30 molecules (green background), all normal modes with a notable Δ*E*_S1_ can be diagnosed from Δ*E*_gap_, with most of these points being close to the Δ*E*_gap_ = Δ*E*_S1_ diagonal (usually *R*^2^ > 0.8). In five further cases, most vibrations with a significant Δ*E*_S1_ can be easily identified from Δ*E*_gap_, but the importance of a single, usually stretching (∼1600 cm^−1^; black arrow in Fig. 1m, n, q and r) vibration is consistently underestimated. SBA does not hold (Fig. 1r and Fig. S2f–h (ESI†); orange background) in very small molecules such as ethene or cyclopropane (Fig. S2g and h, ESI†) due to their tightly bound excitons (*i.e.*, large two-body contributions). Finally, SBA also performs well in molecules where the first excitation does not correspond to a HOMO–LUMO transition (Fig. S3, ESI†).

Our results (Fig. 1 and Fig. S1–S3, ESI†) illustrate that in most small organic molecules only a few (usually < 15%) normal modes are strongly coupled to frontier orbitals. Δ*E*_gap_ can be used to identify the vibration most strongly coupled to frontier orbitals with high confidence (excluding very small systems), and in the majority of cases it correctly identifies vibrations strongly coupled with electronic structure, without resorting to excited-state calculations.

As *E*_bind_ decreases with molecular size,^21^ we expect the SBA to be even more accurate in molecules larger than those investigated here. Therefore, when a description of molecular properties beyond the equilibrium geometry is desired, we suggest using Δ*E*_gap_ to identify normal modes which are likely to affect the electronic structure. Limitations of the SBA are the following: (1) Δ*E*_gap_ only determines the size of the orbital coupling, and the calculation of its asymmetry requires excited state calculations. (2) The values of Δ*E*_gap_ are only valid close to the equilibrium geometry. (3) SBA will typically overestimate Δ*E*_gap_ in cases where frontier orbitals are (near) degenerate (*e.g.*Fig. 1o and p). (4) SBA can be used for transitions involving other orbitals, but this requires a single excited-state calculation (see ESI† Section 4 for details).

Accounting for effects of nuclear motion is particularly important in cases of strong electron-vibration coupling. Usually, in organic molecules the electronic transitions (typically *v*_el_ > 10 000 cm^−1^) are well-separated from vibrational transitions (*v*_*n*_ ≤ 3200 cm^−1^), with much broader and more intense signals. In radical cations of π-conjugated porphyrin oligomers this distinction is less clear, with both electronic and vibrational transitions producing very intense signals in the 1500–6000 cm^−1^ region.^22,23^ These unusually strong vibrational transitions can be detected using infrared (IR) spectroscopy, and they are usually called IR active vibrations (IRAVs). Recently, IRAVs were experimentally found at ∼1330, ∼1550 and ∼2080 cm^−1^ (shaded area in Fig. 2d–f) in the butadiyne-linked porphyrin dimer 1˙^+^ (Fig. 2a), and tentatively assigned to normal modes shown in Fig. 2b. Movement along these IRAVs was attributed to the strong coupling of vibrational modes with the singly occupied molecular orbital (SOMO).^22^ Here, we investigate this coupling in more detail by analysing how normal modes in 1˙^+^ modulate orbital energies (Fig. 2c–f). Using the same density functional as in previous work (LC-ωPBE^22,24^), we calculated the couplings between the HOMO and SOMO energies and normal modes in 1 and 1˙^+^ and compared them with computed dipole moment changes with respect to normal modes (*i.e.* infrared intensities) d*D*/d*Q*_i_ (Fig. 2c–d). Our results show that these frontier orbital energy derivatives are indeed larger in 1˙^+^ than in neutral 1 (Fig. 2c and d), in agreement with previous work. However, we also note that the contribution of orbitals up to SOMO–3 (Fig. 2e) is significant in IRAVs at 1390 cm^−1^ and 2100 cm^−1^, outweighing the contribution of the SOMO.

**Fig. 2:**
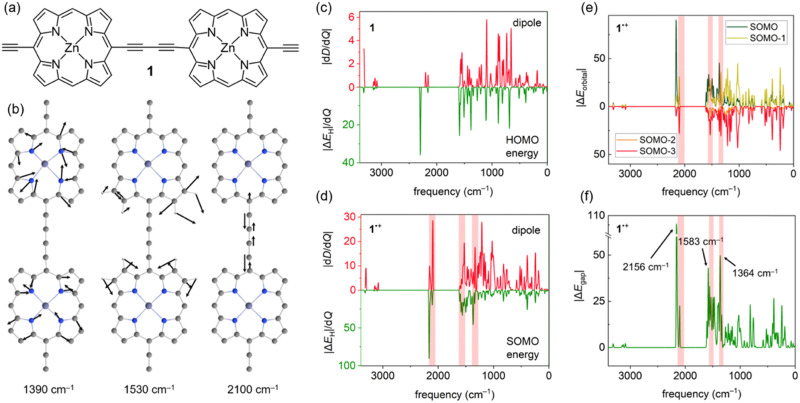
(a) Structure of 1. (b) Calculated IRAVs of 1˙^+^. (c)–(d) Coupling between the computed IR intensities (positive *y* axis) and derivatives of frontier orbital energies (negative *y* axis) in (c) 1 and (d) 1˙^+^. Note the different *y* scales. (e) Contribution of lower-lying orbitals of 1˙^+^. (f) Modulation of the fundamental gap Δ*E*_gap_/d*Q*_i_ in 1˙^+^ with molecular vibrations.

While the dipole moment *D* only depends on the occupied orbitals (within the DFT framework), a proper account of electron-vibration coupling should include the unoccupied orbitals as well. In 1˙^+^, we find that the singly unoccupied molecular orbital (SUMO) is also strongly coupled to movement along several vibrations, resulting in a very large Δ*E*_gap_ (Fig. 2f) at the IRAV positions. Therefore, a large Δ*E*_gap_ may be used as a diagnostic to identify IRAVs and vibrational modes strongly coupled to electronic states, despite being calculated at the equilibrium geometry.

An advantage of using Δ*E*_gap_ instead of the computed IR spectrum is that modes with no IR intensity (d*D*/d*Q*_i_ = 0) due to symmetry can also be IRAVs; indeed, some of the first IRAVs were observed in Raman-active modes.^25^ In case of 1˙^+^, the symmetric stretch of the butadiyne linker at 2156 cm^−1^ (see Fig. 1f and Fig. S4, ESI†) is IR forbidden, but it is strongly coupled to the ground electronic state as it decreases the conjugation between the two porphyrins.

In conclusion, we have presented a simple approach for investigating the influence of individual modes on molecular properties, based on calculating orbital energy derivatives with respect to molecular vibrations. By applying this approach to the Thiel's benchmark set of small organic molecules, we demonstrated that it can in most cases correctly identify the vibrations that are most strongly coupled to the vertical excitation energies by using only a ground-state calculation, which is significantly more efficient than using many excited-state calculations.

Molecules with unusually strong coupling between electronic and vibrational states show intense infrared active bands. Using a porphyrin dimer radical cation as an example, we showed how infrared spectrum can be decomposed into orbital contributions, finding a strong coupling between the IRAVs and the singly occupied molecular orbital (SOMO), but also a significant effect of orbitals up to SOMO–3. Finally, we showed that the sensitivity of frontier orbital energies to nuclear movement can be used to identify IRAVs. We also extended this approach to show how temperature affects the coupling between electronic states in a mixed-valence system.^26^

This work was supported by the ERC (886506 ARO-MAT) and the UKRI (EP/X030075/1). Computational resources were provided by the Cirrus UK National Tier-2 HPC Service at EPCC (https://www.cirrus.ac.uk) funded by the University of Edinburgh and EPSRC (EP/P020267/1).

## Data availability

Code for calculating orbital energy derivatives and building characteristic geometries using eqn (4) is provided at https://github.com/lisa-schroeder/mode-resolved-molecular-properties.

## Conflicts of interest

There are no conflicts to declare.

## Supplementary Material

CC-060-D4CC02066A-s001
