# Supplementary material for: Evaluating the interactions between vibrational modes and electronic transitions using frontier orbital energy derivatives
Source: Chem Commun (Camb). 2024 Jul 2;60(59):7606–9. doi: 10.1039/d4cc02066a (PMC11257084; doi:10.1039/d4cc02066a)
Supplement: CC-060-D4CC02066A-s001 [file CC-060-D4CC02066A-s001.pdf]

Electronic Supplementary Material (ESI) for ChemComm.
This journal is © The Royal Society of Chemistry 2024

Supporting Information for Evaluating the Interactions Between Vibrational Modes and Electronic Transitions Using Frontier Orbital Derivatives

Lisa A. Schröder,[a,b] Harry L. Anderson,[a] Igor Rončević*[a]

[a] Department of Chemistry, Oxford University, Chemistry Research Laboratory, Oxford OX1 3TA, United Kingdom.
[b] Institute of Physical Chemistry, Karlsruhe Institute of Technology, KIT Campus South, Fritz-Haber-Weg 2, D-76131 Karlsruhe, Germany.

(1) Computational details. All calculations were using the B3LYP functional and the def2-SVP basis set[1] using dense numerical grids (grid 4 in Turbomole[2] and int=ultrafine in Gaussian16[3]). All geometries were optimised using tight optimisation criteria (total energy converged to 10^{-7} a.u.). Frequency calculations were done to confirm that the obtained geometries are true minima on the potential energy surface, i.e. that they have no imaginary frequencies. In (B), all computed frequencies were scaled by 0.95. Excited state calculations were done at LR-TD-B3LYP/def2-SVP using Gaussian16.

Our code for both calculating orbital energy derivatives and generating geometries displaced by $\sigma_i(T)$ from *Turbomole*[2] output is available at https://github.com/lisa-schroeder/mode-resolved-molecular-properties. Briefly, the *Turbomole evib*[4] module provides derivatives of orbital energies with respect to atomic movements, while *aoforce* outputs normal modes as linear combinations of atomic movements. The Python script *get_dE_dsigma* uses the output from these modules to calculate the derivative of orbital energies with respect to normal modes. Geometries displaced along a normal mode *i* by the standard deviation of the thermal population $\pm\sigma_i(T)$ at temperature *T* (zero takes zero-point vibration energy into account) can be obtained by using *generate_coords_tmole*.

(2) Static binding approximation (SBA) and the Hubbard model. Let us consider a two-electron two-orbital model described with a Hubbard Hamiltonian, in which *U* and *V* represent the on-site and nearest-neighbour Coulombic interaction, and ε_H and ε_L the HOMO and LUMO energies, respectively (Figure S3). In such a case, the one-electron terms (orange in Figure S3) of the first vertical excitation energy are equal to E_{gap}, while the two-electron terms (blue in Figure S3) correspond to E_{bind} (cf. Figure S3 with eq (1) in main text).

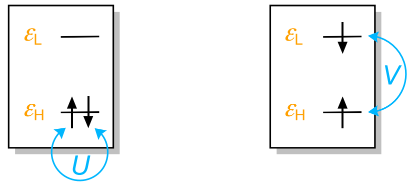

S_0 state S_1 state

$E_{S0} = 2\varepsilon_H + U$ $E_{S1} = \varepsilon_H + \varepsilon_L + V$

$$E_{S1} - E_{S0} = \varepsilon_L - \varepsilon_H + U - V$$
$$E_{S1} - E_{S0} = E_{gap} + E_{bind}$$

Figure S1. First vertical excitation energy in terms of one- (orange) and two-electron (blue) contributions.

When extending the Hubbard model to include electron-vibration coupling, a common approximation is to couple the nuclear degrees of freedom to only the one-electron terms[5-7] and treat *U* and *V* as independent of geometry, which is equivalent to the SBA ($\Delta E_{bind} = 0$, eq (3) in main text).

(3) Performance of the SBA. Figure S2 lists compounds in the Thiel's set in which the $S_0 \rightarrow S_1$ transition can be mostly attributed to the HOMO-LUMO transition, and which are not listed in Figure 1. The SBA shows good performance in the case of first four molecules (Figure S2a–d), mostly good performance for (Figure S2e), while small compounds (S2c,f–h) mostly show poor performance. In the case of tetracene (S2a), anthracene (S2b), and hexatriene (S2b), the first optical transition only corresponds 75–85% to the HOMO-LUMO transition, resulting in the overestimation of ΔE_{gap} relative to ΔE_{bind}. A similar overestimation occurs in benzene (Figure 1o) and zinc porphyrin (Figure 1p).

Please do not adjust margins

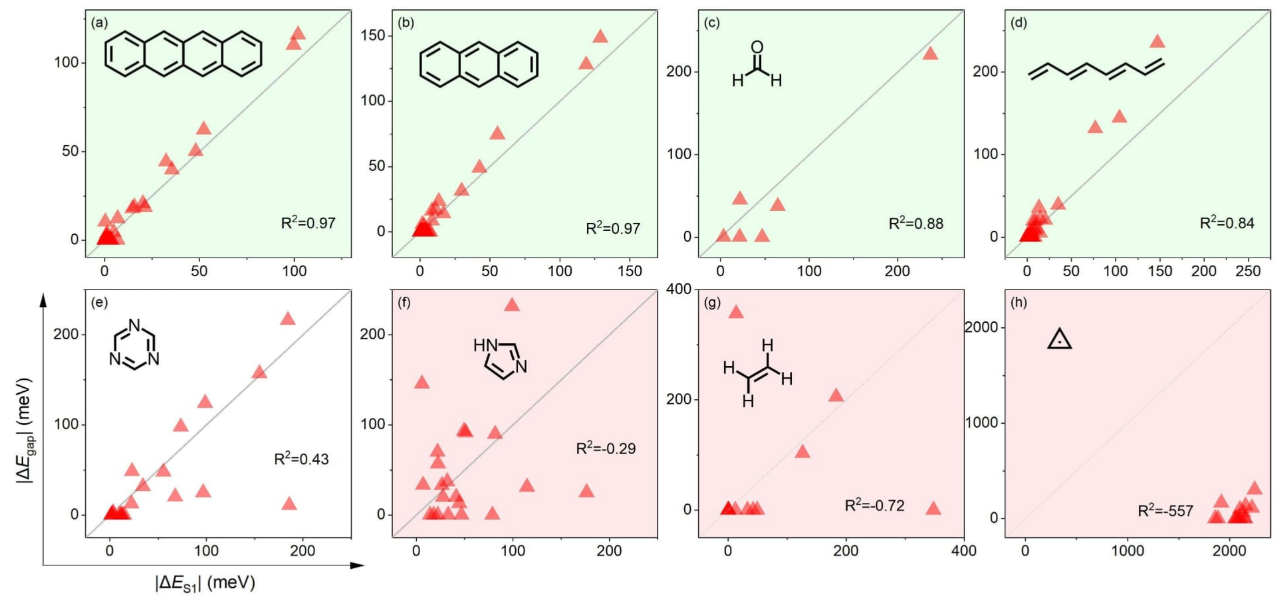

Figure S2. Comparison of ΔE_{gap} (vertical axis) and ΔE_{S1} (horizontal axis) for Thiel's set molecules not included in Figure 1, decomposed by normal modes (triangles).

(4) Beyond HOMO-LUMO transitions. The SBA may be extended to more optical transitions by performing a single excited-state (TD-DFT) calculation, and we recommend doing this for any larger molecule. Such a calculation will characterise the excited states in terms of orbital transitions, enabling us to calculate the ΔE_{gap} for any transition. For example, the first optical transition in adenine (Figure S3a) corresponds to HOMO–1→LUMO, while the second transition (Figure S3e) can be written as roughly 80% HOMO→LUMO + 10% HOMO–2→LUMO+1, and both can be captured using the SBA. Similar good results are obtained in case of thymine (Figure S3b,f) and pyridine (Figure 3c,g), while the results for uracil (Figure 3d,h) are poorer. It is also notable that the SBA performs similarly well for both n→π* and π→π* transitions (cf. top and bottom row in Figure S3). Finally, the SBA is not applicable when the composition of the excited states significantly changes with nuclear motion, as it has no way of predicting how the weights of each contribution change with nuclear movement.

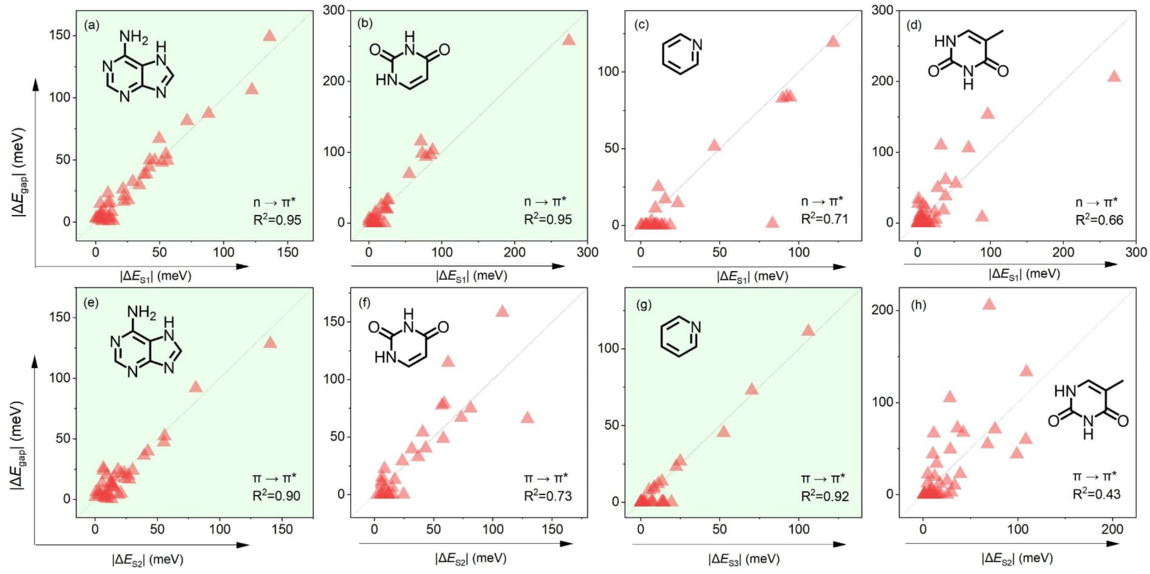

Figure S3. Comparison of ΔE_{gap} (vertical axis) and ΔE_{S1} or ΔE_{S2} (horizontal axis) for Thiel's set molecules not included in Figure 1 nor S2, decomposed by normal modes (triangles). In the case of pyridine (1g), the second optically active transition corresponds to a transition to S3.

This journal is © The Royal Society of Chemistry 20xx

Please do not adjust margins

Please do not adjust margins

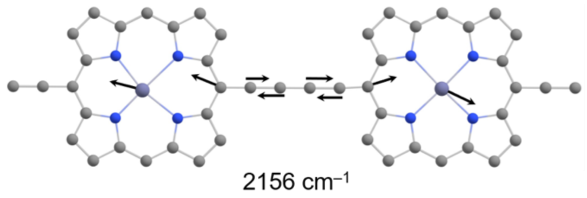

Figure S4. The dipole forbidden vibration in **1**$^{\bullet+}$ strongly coupled to electronic states.

References:

1. F. Weigend and R. Ahlrichs, *Phys. Chem. Chem. Phys.*, 2005, **7**, 3297.
2. S. G. Balasubramani, G. P. Chen, S. Coriani, M. Diedenhofen, M. S. Frank, Y. J. Franzke, F. Furche, R. Grotjahn, M. E. Harding, C. Hättig, A. Hellweg, B. Helmich-Paris, C. Holzer, U. Huniar, M. Kaupp, A. Marefat Khah, S. Karbalaei Khani, T. Müller, F. Mack, B. D. Nguyen, S. M. Parker, E. Perlt, D. Rappoport, K. Reiter, S. Roy, M. Rückert, G. Schmitz, M. Sierka, E. Tapavicza, D. P. Tew, C. van Wüllen, V. K. Voora, F. Weigend, A. Wodyński and J. M. Yu, *J. Chem. Phys.*, 2020, **152**.
3. M. J. Frisch, G. W. Trucks, H. B. Schlegel, G. E. Scuseria, M. A. Robb, J. R. Cheeseman, G. Scalmani, V. Barone, G. A. Petersson, H. Nakatsuji, X. Li, M. Caricato, A. V. Marenich, J. Bloino, B. G. Janesko, R. Gomperts, B. Mennucci, H. P. Hratchian, J. V. Ortiz, A. F. Izmaylov, J. L. Sonnenberg, Williams, F. Ding, F. Lipparini, F. Egidi, J. Goings, B. Peng, A. Petrone, T. Henderson, D. Ranasinghe, V. G. Zakrzewski, J. Gao, N. Rega, G. Zheng, W. Liang, M. Hada, M. Ehara, K. Toyota, R. Fukuda, J. Hasegawa, M. Ishida, T. Nakajima, Y. Honda, O. Kitao, H. Nakai, T. Vreven, K. Throssell, J. A. Montgomery Jr., J. E. Peralta, F. Ogliaro, M. J. Bearpark, J. J. Heyd, E. N. Brothers, K. N. Kudin, V. N. Staroverov, T. A. Keith, R. Kobayashi, J. Normand, K. Raghavachari, A. P. Rendell, J. C. Burant, S. S. Iyengar, J. Tomasi, M. Cossi, J. M. Millam, M. Klene, C. Adamo, R. Cammi, J. W. Ochterski, R. L. Martin, K. Morokuma, O. Farkas, J. B. Foresman and D. J. Fox, *Journal*, 2016.
4. M. Bürkle, J. K. Viljas, T. J. Hellmuth, E. Scheer, F. Weigend, G. Schön and F. Pauly, *Phys. Status Solidi B*, 2013, **250**, 2468-2480.
5. W. Barford, *J. Phys. Chem. Lett.*, 2023, **14**, 9842-9847.
6. D. Manawadu, T. N. Georges and W. Barford, *J. Phys. Chem. A*, 2023, **127**, 1342-1352.
7. W. Barford and R. J. Bursill, *Phys. Rev. B*, 2006, **73**, 045106.

This journal is © The Royal Society of Chemistry 20xx

Please do not adjust margins
